# Mechanical Characterization of One-Headed Myosin-V Using Optical Tweezers

**DOI:** 10.1371/journal.pone.0012224

**Published:** 2010-08-18

**Authors:** Tomonobu M. Watanabe, Atsuko H. Iwane, Hiroto Tanaka, Mitsuo Ikebe, Toshio Yanagida

**Affiliations:** 1 WPI Immunology Frontier Research Center, Osaka University, Suita, Osaka, Japan; 2 Department of Physiology, University of Massachusetts Medical School, Worcester, Massachusetts, United States of America; 3 Soft Biosystem Group, Laboratories for Nanobiology, Graduate School of Frontier Biosciences, Osaka University, Suita, Osaka, Japan; 4 Kobe Advanced ICT Research Center, National Institute of Information and Communications Technology, Kobe, Japan; CNRS, France

## Abstract

Class V myosin (myosin-V) is a cargo transporter that moves along an actin filament with large (∼36-nm) successive steps. It consists of two heads that each includes a motor domain and a long (23 nm) neck domain. One of the more popular models describing these steps, the hand-over-hand model, assumes the two-headed structure is imperative. However, we previously succeeded in observing successive large steps by one-headed myosin-V upon optimizing the angle of the acto-myosin interaction. In addition, it was reported that wild type myosin-VI and myosin-IX, both one-headed myosins, can also generate successive large steps. Here, we describe the mechanical properties (stepsize and stepping kinetics) of successive large steps by one-headed and two-headed myosin-Vs. This study shows that the stepsize and stepping kinetics of one-headed myosin-V are very similar to those of the two-headed one. However, there was a difference with regards to stability against load and the number of multisteps. One-headed myosin-V also showed unidirectional movement that like two-headed myosin-V required 3.5 k_B_T from ATP hydrolysis. This value is also similar to that of smooth muscle myosin-II, a non-processive motor, suggesting the myosin family uses a common mechanism for stepping regardless of the steps being processive or non-processive. In this present paper, we conclude that one-headed myosin-V can produce successive large steps without following the hand-over-hand mechanism.

## Introduction

The myosin super family consists of motor proteins that move and/or generate force unidirectionally along actin filaments in order to regulate a vast number of essential cellular processes including muscle contractions, vesicle transport, and cell division [1], [2]. In order to reveal the mechanism for force generation, many researchers have observed myosin's single molecular mechanical properties [3], [4]. Class V myosin (myosin-V), although an unusual myosin in that it generates large successive (∼36 nm) steps, much larger than the ∼5 nm steps taken by myosin-II during muscle contraction, is quite popular for such studies [5], [6]. Myosin-V has two heads, each of which consists of a motor domain and a long neck domain which influences the stepsize [7], [8]. Based on these structural features, a “hand-over-hand” model has been proposed to explain its unidirectional and successive large steps [9]. Novel fluorescent techniques that offer nm resolution and the angle of the fluorophore have affirmed this model [9]-[12]. A central premise for multiple successive large steps according to the model is that the two-headed structure is indispensable. This has been reaffirmed by previous studies that have shown myosin-V subfragment 1, a one-headed version of myosin-V, fails to make successive steps [13], [14] and the argument that the two heads are necessary because processive stepping is regulated by the internal strain between them [15]–[18].

However, other studies have challenged this conclusion. We have observed that one-headed myosin-V included into headless myosin-II cofilaments can also generate successive 36 nm steps [19]. We have also incorporated one-headed myosin-V smooth muscle myosin rod chimeras (M5SH) into myosin rod filaments, allowing us to determine the orientation of the actomyosin interaction, finding that the stepsize of myosin-II depends on the angle between the cofilament and the actin filament [20]. Optimizing this angle enables one-headed myosin-V to take successive multiple 36 nm steps [19]. Furthermore, other one-headed myosins have been found to move successively including myosin-VI [21] and myosin-IX [22]. Here, we compared the motility properties between M5SH and M5DH, a two-headed myosin-V chimera. Overall, the data suggest that one-headed myosin-V can produce successive steps, which implies that the hand-over-hand mechanism is not the only mechanism used by myosin to achieve processive movement, although it may be the preferred one.

## Results

### Single molecular measurements of M5SH and M5DH using optical tweezers

**Figure 1:**
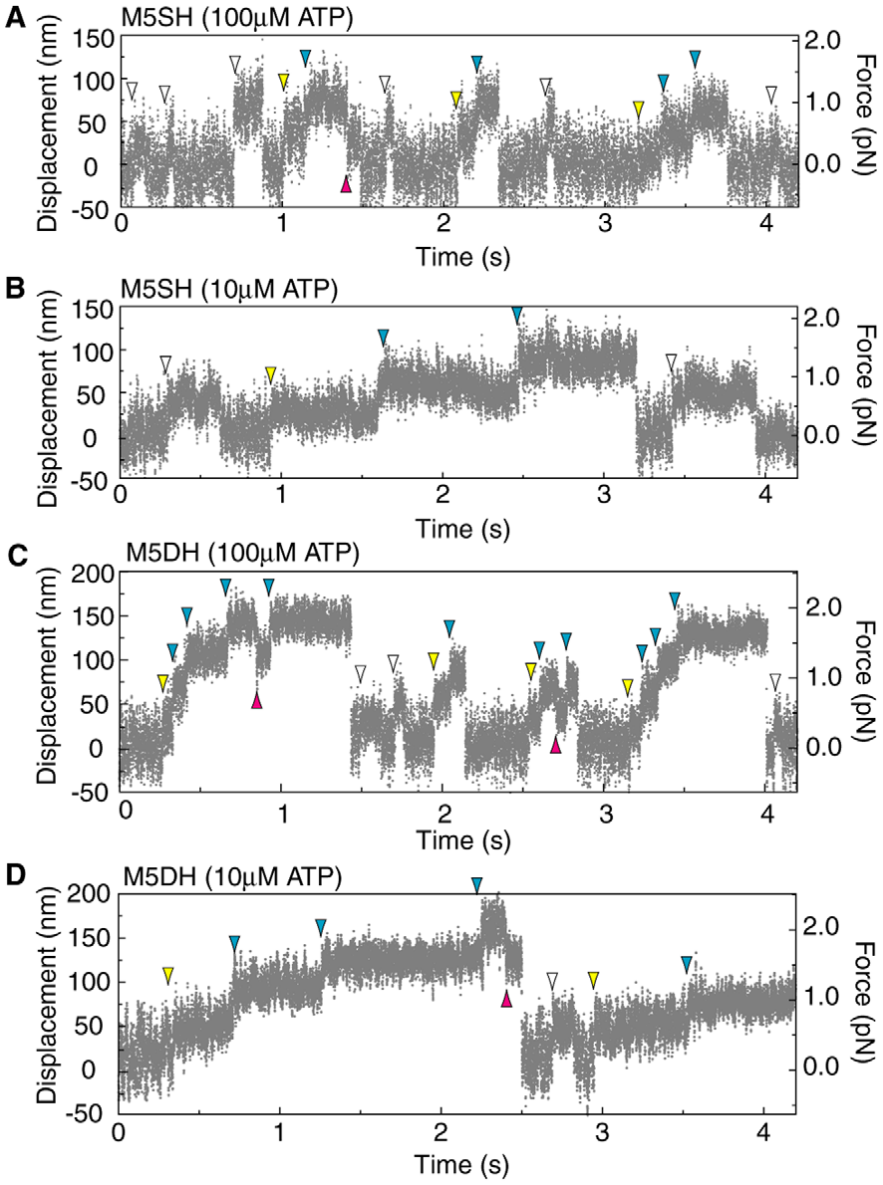
Mechanical steps. Typical traces of time courses for M5SH steps at 100 µM ATP (A) and 10 µM ATP (B), and for M5DH at 100 µM ATP (C) and 10 (D) µM ATP. Gray dots represent raw data. White arrowheads show non-successive strokes; yellow arrowheads, first strokes during the successive-multisteps; blue arrowheads, successive steps; red arrowheads, backward steps. Medium: 120 mM KCl, 5 mM MgCl_2_, 1 mM EGTA, 0.2 mg/ml calmodulin and 20 mM Hepes (pH 7.8). Trap stiffness, 0.02–0.025 pN/nm. Temperature, 25°C.

Movements of M5SH and M5DH along an actin filament were observed by optical trap nanometry, as previously reported [19]. We detected successive large steps for both M5SH and M5DH when mixed with headless myosin cofilaments (Fig. 1). Cofilaments were adsorbed onto the surface of a pedestal made on a quartz glass surface [19], [20], [23]. The long cofilaments allowed us to easily find the location of the M5SHs and M5DHs and also manipulate the actin filaments to make a favorable angle with the heads (65∼80° for M5SH; 45∼60° for M5DH), otherwise, the probability of successive stepping markedly decreased [19]. Mechanical steps of single M5SHs and M5DHs at 100 and 10 µM ATP are shown in Fig. 1 *A*–*D*. The traces of the bead displacements consisted of successive multisteps (Fig. 1 *yellow and blue arrowheads*), although the displacements sometimes developed in a single-step fashion (Fig. 1 *white arrowheads*). For M5SH cofilaments, 55 cofilaments among 509 cofilaments interacted with the actin filament, with 37 of these generating successive multisteps (Table 1). This probability (37/509 = 0.07) corresponds to that in our previous report in which we discussed the possibility that a cofilament includes a single M5SH molecule [19]. For M5DH cofilaments, 20 of the 166 cofilaments interacted with the actin filament, all of which showed multiple steps. From these results, we classified three step types: non-successive strokes (Fig. 1 *white arrowheads*), first strokes during multisteps (Fig. 1 *yellow arrowheads*) and successive steps (Fig. 1 *blue arrowheads*). The more common direction of movement was denoted as “forward.” Backward steps during successive multisteps were sometimes observed (Fig. 1 *red arrowheads*).

**Table 1:** Summary of experiments.

		M5SH	M5DH
No. of cofilaments tested		509	166
No. of cofilaments interacting with actin filaments		55	20
No. of cofilaments showing successive steps		37	20
Non-successive steps	Stepsize (nm)	23±20	22±16
	Dwell time(s)*	0.45,	0.49,
		0.10	0.10
	Vmax (s^−1^)	11	N.D.
	Km (µM)	36	N.D.
First step during successive steps	Stepsize (nm)	20±16	22±17
	Dwell time(s)*	0.50,	0.44,
		0.10	0.11
	Vmax (s^−1^)	11	N.D.
	Km (µM)	28	N.D.
Successive steps	Stepsize (nm)†	32±13	34±15
		−36±17	−30±13
	Dwell time(s)*	0.40,	0.39,
		0.11	0.18
	Vmax (s^−1^)	10	N.D.
	Km (µM)	30	N.D.
Load dependency of directionality	Energy difference (k_B_T)	3.7	3.4
	Characteristic distance (nm)	5.7	4.5
	Stall force (pN)	0.8	2.2
Successivity		0.28	0.5

All errors are SD.

*100 µM ATP, 10 µM ATP, respectively.

† Forward and backward, respectively.

### Step/stroke size of M5SH and M5DH

**Figure 2:**
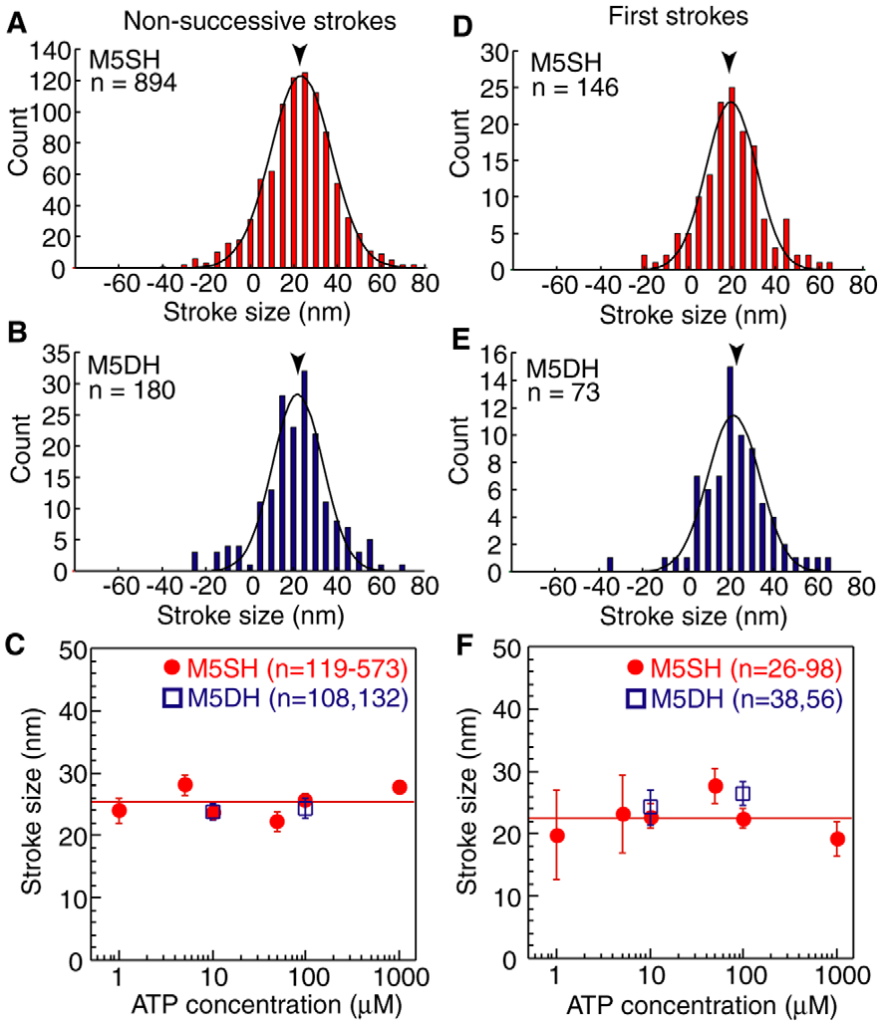
Size of non-successive stokes and first strokes. (A, B) Histogram of non-successive strokes for M5SH (A) and M5DH (B) in 10 µM ATP, respectively. Lines are Gaussian distribution fits. Arrowheads indicate mean stroke sizes of 23±20 nm (A) and 22±16 nm (B) (mean ± S.D). (C) ATP dependency of size for non-successive stroke. Red circles and blue squares indicate data for M5SH (n = 119–573) and M5DH (n = 108–132), respectively. Red line is the average M5SH stepsize, 25.3 nm, across all ATP concentrations. (D, E) Histogram of first strokes for M5SH (D) and M5DH (E) in 10 µM ATP. Lines are Gaussian distribution fits. Arrowheads indicate the mean stroke sizes, 20±16 nm (D) and 22±17 nm (E). (F) ATP dependency of the size of first stroke. Red circles and blue squares indicate data for M5SH (n = 26–98) and M5DH (n = 38–56), respectively. Red line is the average M5SH stroke size across all ATP concentrations. Error bars in C and F indicate standard errors.

One mechanical property of frequent interest in myosin motors is the size of the stroke and step. We analyzed the size of non-successive strokes (Fig. 2 *A–C*) and first strokes (Fig. 2 *D–F*) to investigate whether they were different. However, the size of individual first and non-successive strokes cannot be determined directly because the actual start positions of the steps are random due to the Brownian motion of the beads [24]. Therefore, we determined the mean size of these strokes from a histogram of stroke sizes based on the plateau position after each stroke. The mean stroke sizes of non-successive strokes for M5SH and M5DH were 23±20 nm (n = 894) and 22±16 nm (n = 180), respectively (Fig. 2 *A* and *B*). Those of the first strokes were 20±16 nm (n = 164) and 22±17 nm (n = 73), respectively (Fig. 2 *D* and *E*). The values for non-successive M5SH strokes and the first strokes of M5DH were consistent with other groups [13], [14]. Furthermore, here we found no differences in the sizes of first strokes and non successive strokes for either M5SH or M5DH. Finally, the stroke sizes were independent of ATP concentration buffer (Fig. 2 *C* and *F*).

**Figure 3:**
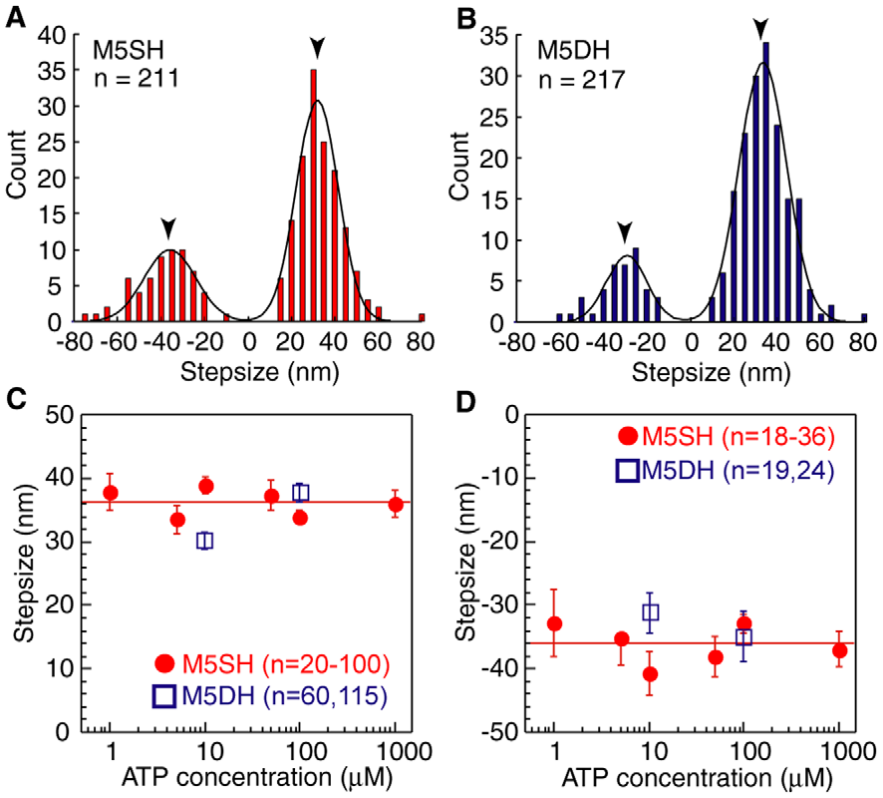
Size of successive steps. (A, B) Histogram of successive steps for M5SH (A) and M5DH (B) in 10 µM ATP. Lines are Gaussian distribution fits. Arrowheads indicate peak values, 32±13 and −36±17 nm (A), and 34±15 and −30±13 nm (B) (mean ± S.D), respectively. (C, D) ATP dependency for forward (C) and backward (D) stepsize during successive steps. Red circles and blue squares indicate M5SH (C, n = 20–100; D, n = 18–36) and M5DH (C, n = 60–115; D, n = 19–24), respectively. Red lines are average stepsizes for forward, 36.3 nm, and backward, −36.1 nm, steps. Error bars in C and D indicate standard errors.

Since we could observe successive multisteps for M5SH and M5DH using the cofilament assay system, we next analyzed stepsizes of successive steps for each (Fig. 3). These stepsizes were defined as the distance between the forward position averaged for 50 ms after the stepping point and the backward position averaged for 50 ms before the stepping point (Fig. 1, distance between consecutive *blue and red arrowheads*). The histogram of stepsizes for M5SH successive steps fit to a Gaussian distribution with a forward value of 32±13 nm and backward value of 36±15 nm. Both forward and backward steps for M5SH were the same as those reported for myosin-V HMM [5], [6], [13]. The stepsizes of successive steps were also independent of ATP.

### Dwell time distributions

**Figure 4:**
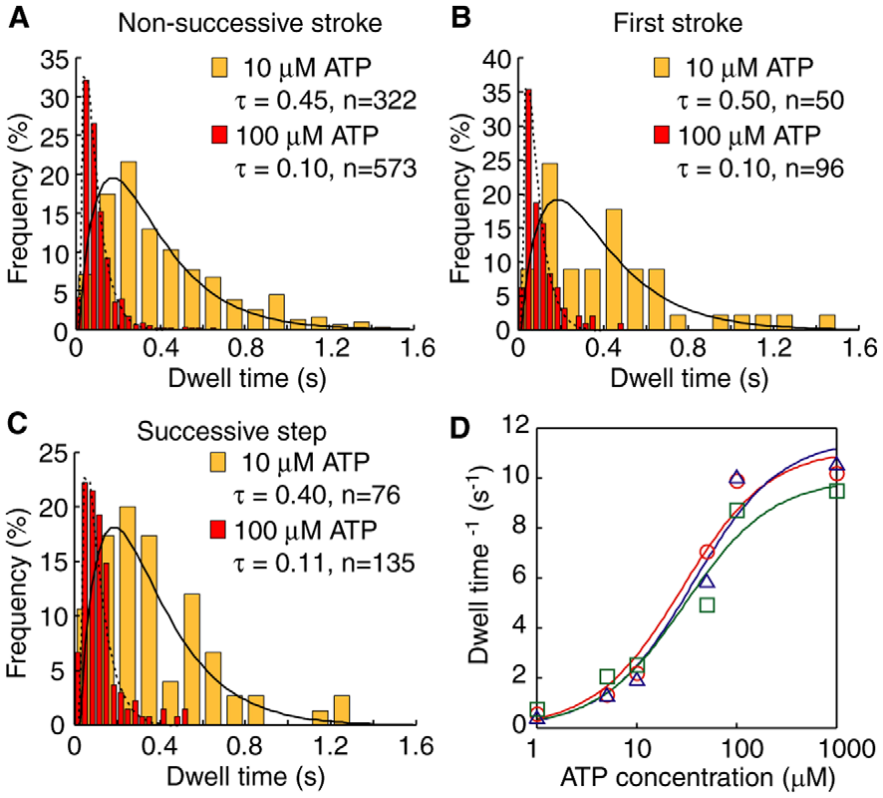
Dwell time of M5SH. (A, B, C) Histograms of dwell times for non-successive strokes (A), first strokes (B) and successive steps (C) for M5SH. The successive step was excluded from the analysis for first strokes. Orange bars indicate data for 10 µM ATP; red, 100 µM ATP. Lines indicate fitting results to the double exponential curve *f*(*t*) = (*k*_1_·*k*_2_/(*k*_1_−*k*_2_)}/(exp(*k*_2_ *t*)−exp(*k*_1_ *t*)} (solid, 10 µM ATP; broken, 100 µM ATP). The mean dwell times (τ) were calculated as τ = [1/*k*_1_+1/*k*_2_]. (D) ATP concentration dependency of dwell times (red circles, non-successive strokes; blue triangles, first strokes; and green squares, successive steps). The dwell time represents ATPase rate per second assuming each step corresponds to a single ATP hydrolysis event. Solid lines indicate fitting results assuming monophasic Michaelis-Menten kinetics. Vmax and Km were 11 s^−1^ and 28 µM for non-successive strokes, 11 s^−1^ and 36 µM for first strokes, and 10 s^−1^ and 30 µM for successive steps, respectively.

To investigate the relationship between ATP hydrolysis and step/stroke generation, we estimated the dwell time (time until detachment for non-successive strokes or the time for the next step following first strokes or successive steps) (Fig. 4). Assuming non-successive strokes are the result of the myosin head detaching from actin independent of ATP hydrolysis, there should exist a difference in dwell times between non-successive strokes and first strokes. Fig. 4 shows the dwell times of the two for M5SH (Fig. 4 *A* and *B*). The distributions of the dwell times at 10 µM ATP show double exponential behavior, indicating that both were generated by a two rate-limiting transition [6]. Increasing ATP concentration made the stepping faster (Fig. 4 *A* and *B*, *orange bars*), while the reciprocal plot of mean dwell times for both step types fit well to monophasic Michaelis-Menten kinetics (Fig. 4 *D*). Vmax and Km for non-successive strokes were respectively 11 s^−1^ and 28 µM (Fig. 4 *D circle*), while those for the first stroke were respectively 11 s^−1^ and 36 µM (Fig. 4 *D triangle*). Unlike our assumption, neither the dwell times nor the Michaelis-Menten kinetics showed any differences between the two step types, indicating that detachments needed energy from ATP hydrolysis in the same manner as taking a second step. The reciprocal plot of dwell times for successive steps showed the same distribution and same Michaelis-Menten kinetics (Vmax of 10 s^−1^ and Km of 30 µM) (Fig. 4 *C* and *D*). These Vmax values are similar to those biochemically obtained in solution [25], [26] and from myosin-V HMM studies using laser trap nanometry [5], [6], suggesting that each step corresponds to a single ATP hydrolysis event.

**Figure 5:**
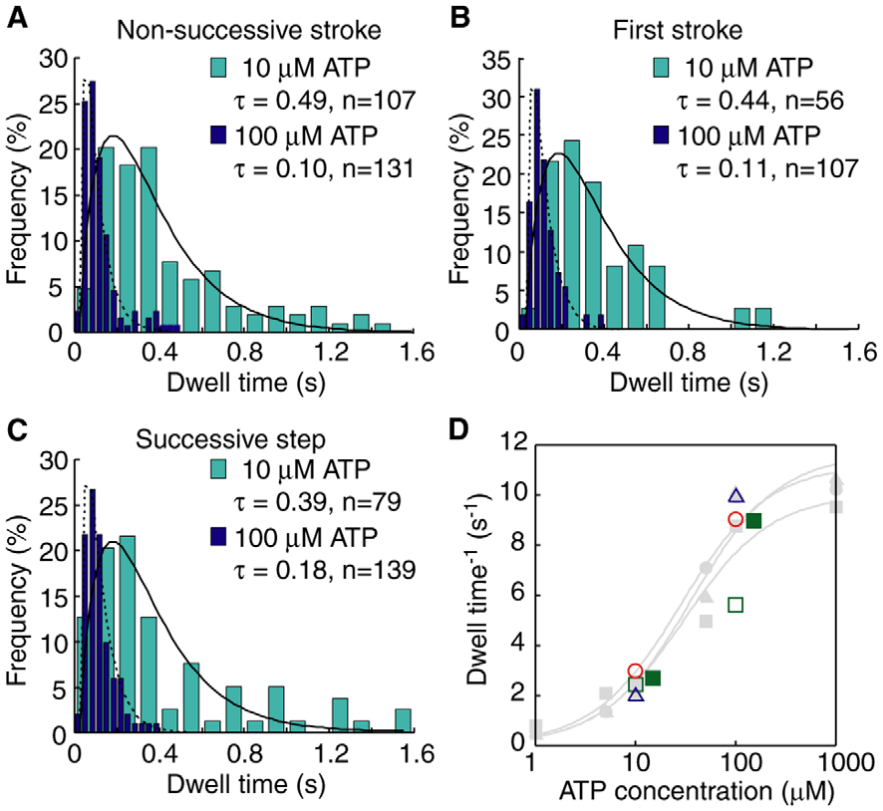
Dwell time of M5DH. (A, B, C) Histograms of dwell times for non-successive strokes (A), first strokes (B) and successive steps (C) for M5DH. The successive steps were excluded from the analysis for first strokes. Cyan bars indicate data for 10 µM ATP; blue, 100 µM ATP. Lines indicate fitting results with a double exponential curve (solid, 10 µM ATP; broken, 100 µM ATP). τ are the mean dwell times. (D) ATP concentration dependency of dwell times (red circles, non-successive strokes; blue triangles, first strokes; and green squares, successive steps). The green solid square was obtained from data under a low load (<1.5 pN). Gray symbols and lines indicate M5SH results (see Fig. 4 *D*).

We also analyzed the dwell times of M5DH non-successive, first strokes and successive step (Fig. 5). Assuming that the two heads generated steps independently, the dwell times for M5DH should be half that of M5SH. The distributions of the dwell times for all showed the same properties and were dependent on ATP concentration. Each dwell time under low load (<1.5 pN) in the presence of 10 or 100 µM ATP could be plotted on the same monophasic Michaelis-Menten kinetics found in the M5SH data. Therefore, successive steps by two-headed myosin-V are generated via the same chemical-physical energy transition process as one-headed ones. This result suggests that the two heads in M5DH alternately generates steps using cooperativity. One additional point is that the dwell time at 10 µM ATP (∼0.4 s) was a little higher than that previously reported using myosin-V HMM (0.28 s) [5]. However, since M5DH used not native coiled-coil but S-2 fragments of smooth muscle myosin-II, the hand-over-hand coordination might be disrupted in a manner that delays ADP release, which in turns increases the dwell time.

### Force dependency of stepsize, dwell time, and unidirectionality of successive steps

**Figure 6:**
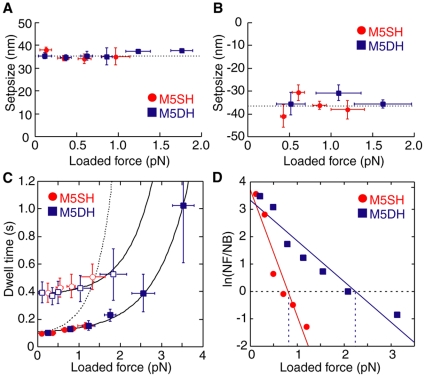
Force dependencies for stepsize, dwell time, and unidirectionality. (A, B) Force dependencies of forward (A) and backward (B) successive steps. Red circles indicate data for M5SH (n = 15–59 in A, 9–18 in B), blue squares indicate data for M5DH (A, n = 19–31; B, n = 12–16). Broken lines are average values (A, 35.4 nm; B, −36.5 nm). All data were obtained at 10 µM ATP. (C) Force dependencies for dwell time of M5SH (red circles) and M5DH (blue squares). Each point represents the average dwell time at 100 µM ATP (solid: n = 15–59 for M5SH; 20–67 for M5DH) and 10µM (open: n = 30–37 for M5SH; 18–37 for M5DH). The curves were obtained from equation (1). (D) Ratio of forward to backward movements for M5SH (red circles: n = 14–47) and M5DH (blue squares: n = 36–47). The solid lines were obtained from equation (3). NF is the number of forward steps; NB, backward steps.

Optical trapping nanometry has been used to observe the stepping of individual motors against constant loads [5]. For example, the stepsize of one-headed myosin-VI was seen to depend on the load generated by the laser trap, which was not the case for two-headed myosin-VI [21], [27]. Here we examined the dependency of stepsize in successive M5SH steps on load (Fig. 6 *A* and *B*, *red circles*). Plots were limited to 1.5 pN because M5SH could not take steps at higher loads. Successive M5SH steps were ∼36 nm and unaffected by load regardless of being forward or backward, much like myosin-V HMM [28]. For comparison, we analyzed M5DH steps and found their stepsize did not depend on load either (Fig. 6 *A* and *B*, *blue squares*).

To further understand the role of the two-headed myosin-V structure, we next compared the force-dependency of the dwell time for successive steps between M5SH and M5DH. Assuming that the myosin head chemo-mechanical cycle consists of a series of biochemical steps and a single load-dependent mechanical transition, the mean dwell is given by a Boltzmann-type relation;
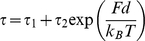
(1)where τ_1_ represents the load-independent transition, *d* is the characteristic distance, which describes the load dependency, τ_2_ represents the load-dependent transition for the bead to diffuse d at zero load, k_B_ is the Boltzmann constant, and T is absolute temperature [29], [30]. Plots for M5SH and M5DH were fitted with the same single exponential curve, which was very similar to that used to fit myosin-V HMM (Fig. 6 *C*) [5]. Fitted values in 100 µM ATP for τ_1_, τ_2_ and *d* were 98 ms, 7.7 ms and 5.7 nm, respectively, while τ_1_/τ_2_ (127.3) was similar to that obtained for myosin-V HMM by another group [5]. Decreasing the ATP concentration lengthened τ_1_ (0.38 s for 10 µM ATP), but changed neither τ_2_ (9.8 ms) nor *d* (6.4 nm), indicating that the ATP dependency of the dwell time was independent of load. The fact that there were no observed differences between the load dependency for M5SH and M5DH dwell times indicates that the external force was not divided into each of the two M5DH heads; rather only the stepping head experienced load regardless of the number of heads. Otherwise, *d* for M5SH would be half that of M5DH (Fig. 6 *C broken line*).

Myosin-V stepped not only forward but also backward as mentioned above (Fig. 1 *red arrowheads*). The energy difference between the forward and backward movements by M5SH and M5DH could be estimated by the load dependence of the respective stepping direction when assuming an asymmetric potential like that previously reported for kinesin [31]. After removing detachments, we counted the number of forward and backward steps. The ratio of forward to backward steps was plotted as a function of load (Fig. 6 *D*) as follows:
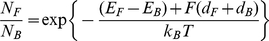
(2)

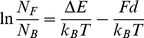
(3)where *N_F_* and *N_B_* are the number of forward and backward steps, respectively, *E_F_* and *E_B_* are the energetic heights of the barrier maximum at zero load, *d_F_* and *d_B_* are the characteristic distances against load *F*, Δ*E* = *E_B_*−*E_F_* is the energy difference, and *d* = *d_B_*+*d_F_* is the characteristic distance that represents the load dependency [31], [32]. The fitting results from equation 3 gave Δ*E* = 3.7 k_B_T and *d* = 19 nm for M5SH (Fig. 6 *D*, *red*). Interestingly, the Δ*E* for M5DH (3.4 k_B_T) was similar to that of M5SH while the *d* of M5DH (6.2 nm) was 3-fold smaller than that of M5SH. The stall force (*N_F_* = *N_B_*), which is the maximum force at zero velocity, was estimated to be 0.8 pN for M5SH and 2.2 pN for M5DH. The similar Δ*E* indicates the two-headed structure did not contribute to unidirectionality (*N_F_*/*N_B_* ratio) whereas the small *d* for M5SH suggests the two-headed structure is more stable against load.

### Successivity of M5SH and M5DH

**Figure 7:**
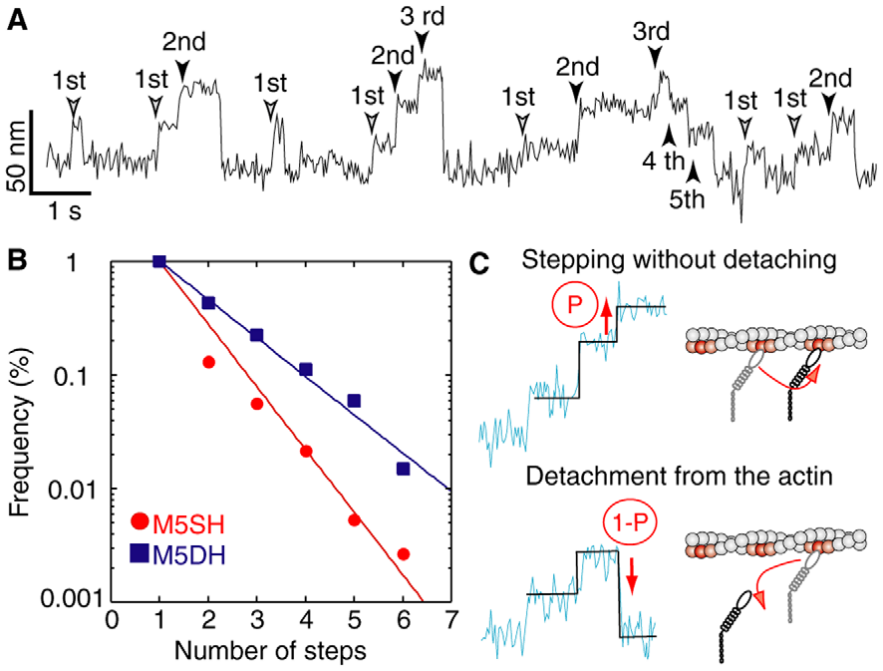
Probabilities of successive steps. (A) How to count successive steps. Backward steps were included within successive steps. Total number of steps before detachment make up ‘number of steps’ in B. (B) Number of steps by M5SH (red circles; n = 373) and M5DH (blue squares; n = 182) in 100 µM ATP. Solid lines are the fitting results with a single exponential curve. (C) Explanation of probability P. Myosin heads generated the next successive step with a probability of P and detached with that of (1−P).

Finally, we estimated the average number of successive steps to investigate whether the two-headed structure stabilizes the long travel done by myosin-V (Fig. 7). In general, almost immediately after ATP binds to a myosin head, the head detaches from actin. In order to achieve multiple steps, M5SH must conform to the strong binding state in order to step without detaching. We defined this probability as successivity. In this definition, non-successive strokes are those that take one step and then detach (Fig. 7 *A*). The data in Fig. 7 *B* were fit with a single exponential curve, *f*(n) = exp(P(n−1)}, where P is the successivity and n is the number of total steps before detaching (Fig. 7 *C*). The values of P were 0.28 for M5SH and 0.50 for M5DH. The number of successive multisteps by M5SH before detaching (*N_enc_*) is described as follows
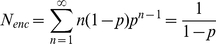
(4)where *p* is the successivity. *N_enc_* was calculated to be 1.4. This indicates that successive steps by M5SH cannot be observed in bio-chemical assays in solution and/or single molecular imaging using fluorescent dyes. Furthermore, *N_enc_* for M5DH was 2, which translates to displacements of about 80 nm, far less than the thousand nm displacements done by native myosin-V [6]-[13]. The successivity of M5DH calculated using the successivity of M5SH (0.28) is 0.52 = (1−0.28)^2^, which nearly equals the experimental value (0.50), indicating that the two-headed structure did not contribute the successivity. There were no differences in successivity between 10 and 100 µM ATP for either M5SH or M5DH (data not shown).

## Discussion

Here we found that the mechanical characteristics of one-headed myosin-V (M5SH) were very similar to that of a two-headed myosin-V chimera (M5DH), suggesting that one-headed myosin-V like its two-headed counterpart can achieve successive steps. Various parameters determined in the present study are summarized in Table 1. Although other groups have failed to observe successive steps by one-headed myosin-V [13], [14], we have reasoned that this is due to the one-head's successivity being dependent on the angle of the actomyosin interaction [19]. We also did a single molecular motility assay in combination with total internal refraction microscope [3] to investigate the single-headed myosin steps (Figure S1). While GFP (green fluorescent protein) fused to double-headed myosin-V moved unidirectionally along an actin filament with a 250 nm travel distance, single headed myosin-V proved incapable of displacing such long distances. The single bead trapping assay [6] and three beads dumbbell assay [5] also failed to observe successive steps by single-headed myosin-V. The cofilament assay we performed here might create preferable conditions for such steps by constraining M5SH, most likely a consequence of myosin-V's rigid neck region and the helical structure of the actin filament. Experiments using single-headed constructs of other myosins that lack a cofilament system like myosin-VI and myosin-IX have also observed successive steps, but these myosins have much more flexible necks than myoins-V likely making the angle dependency between the myosin and actin in those circumstances negligible [21], [22].

One report has suggested that the stepsize of myosin-V depends on the neck region length [33]. Furthermore, successive large steps have to date been explained by a hand-over-hand model where tilting of the long neck domain of the lead head biases the Brownian motion of the rear head forward [9]–[13], [34]. The rear head detaches upon ATP binding and rapidly moves forward ∼72 nm to bind to actin [9], [10], [12]. That means the lead head generates the step direction by titling its neck such that the rear head rebinds at a forward actin binding site. According to this model, the stepsize and neck length are proportional and one-headed myosin are incapable of successive steps. However, we have previously reported that short-necked myosin-V can produce successive large steps, challenging this model [23]. So too, of course, do our results here. Ultimately, an alternative model is needed to explain one-headed successivity. We assume that M5SH when in a weak binding state does not completely dissociate from actin but rather diffuses along the actin filament using Brownian energy. In the double trapping nanometry (dumbbell assay), preferred binding sites on an actin filament appear every 36-nm along the helix [35]. Myosin-V binds to these preferred sites while diffusing along the filament. A change in strain on the head causes Pi release, which conforms the head into a strong binding state [28], [36].

The size and kinetics (dwell time) of the M5SH steps/strokes are consistent with M5DH and myosin-V HMM [5], [6], [13], [28]. To confirm therefore that the M5SH results were actually due to single heads and not two heads that were within such proximity that they could not be resolved by our equipment, we examined the properties of M5DH. While the stepsize and dwell time were the same, the load dependence between M5SH and M5DH was different. If we assume that successive steps by M5SH were actually due to two adjacent heads taking non-successive strokes, then the stepsize and dwell time for M5SH should be half that of M5DH and no differences in load dependence would be observed. Therefore, the results indicate that the observed mechanical activities of M5SH were due to one myosin-V head while M5DH functioned with some level of cooperativety between the two heads to prevent the two heads from simultaneously stepping, which would result in detachment. This may also be in part a consequence of our cofilament assay system, may generate two-headed structures that are geometrically constrained when interacting with actin in a manner that reduces the likelihood of successive steps.

M5SH and M5DH showed no differences between the dwell time force dependencies (Fig. 6 *C*) or between the energy barriers for the direction of movement (Fig. 6 *D*). These results lead us to think that only one of the two heads in M5DH strongly interacts with the actin filament, which would enable large steps without applying the hand-over-hand mechanism. It should be noted that M5DH was dimerized with the myosin-II S2-fragment, not the coiled-coil region of myosin-V, which may account for a different mechanism. Still, while M5DH and M5SH shared some properties, they differed in other important ones. For example, M5DH had a higher stall force (Fig. 6 *D*) and lower probability for detachment (Fig. 7). This was also seen when comparing two-headed and one-head muscle myosin-II [37], suggesting that the dimerized heads in our M5DH cooperate in a manner more similar to muscle myosin-II than native myosin-V. Therefore, it is possible that the coiled-coil region and/or the long neck length of native myosin-V is critical for stepping in a hand-over-hand manner, which would achieve long travel distances. This would explain why the distance travelled as estimated from the M5DH successivity (0.5) was shorter (80 nm) than that seen in myosin-V HMM (Fig. S1). Another point of interest is the bias between forward and backward steps, a point we discuss in more detail below, which was 3.5 k_B_T and is similar to that estimated for myosin-II (2–3 k_B_T) [38]. This may suggest that despite different behavior, the mechanism for directional bias might be the same among different myosins.

The process for M5SH steps following the strong binding state is thought to be ATP binding dependent, as there were no differences in the stepping kinetics for non-successive strokes, first strokes and successive steps (Fig. 4). Since the cofilament assay constrains the geometry of the acto-myosin interaction, it is possible that an observed successive step was actually a quick detachment and reattachment by the myosin-V head. Even if this is the case, for a second successive to be the same, the probability of a second reattachment 36 nm from the previous spot was estimated to be approximately 38% by using the trap stiffness (0.02 pN/nm) and Gaussian distribution of the trapped bead position. Moreover, for a third step successive step, the probability would be 3.8%, one-tenth the experimental value (30%). Therefore, successive M5SH steps are not the result of a quick detachment and reattachment. Assuming a myosin-V head diffuses along an actin filament when weakly bound, if the myosin-head finds an adjacent actin binding site during this diffusion period, it can release Pi and step forward; if not, it detaches. Under this assumption, M5SH can bind to 3 sites: forward, backward and the same position. Since the bias between forward and backward steps was 3.5 k_B_T, the probabilities of the three bindings are 83.0% (forward), 14.6% (same position) and 2.4% (backward). Taking into consideration successivity (Fig. 7), the probabilities that second forward or backward steps occur are 25% ( = 0.3×83%) and 0.7% ( = 0.3×2.4%), respectively. The remaining 74.3%, including the probability for binding to the same position (4.3% = 0.3×14.6%), is the probability of nonsuccessive strokes.

A possible model for explaining how M5SH develops its successive large steps is shown in supplemental Fig. S2. M5SH in the ADP.Pi state diffuses back and forth over the actin filament via thermal stretching of its neck region and repeated dissociations and associations to an actin filament. When releasing Pi to take a strong binding state, the head tilts its long neck forward (23 nm stroke) and then releases ADP. When a subsequent ATP binds to the head, the head again diffuses along the actin filament. This time, should the head completely dissociate from actin, a non-successive stroke is observed. If, however, the head diffuses to a favorable binding site (13 nm forward), the head strains backward and thus accelerates strong binding in the forward target region based on the search-and-catch model [36]. After rebinding, another 23 nm stroke occurs, enabling successive steps to be observed.

The behavior of M5DH can be explained by a similar model shown in supplemental Fig. S3. One head strongly interacts with actin while the other weakly interacts. Therefore, only one head waiting for ATP binding senses a load, which explains why we saw no difference in the force dependency on dwell time between M5SH and M5DH (Fig. 6C). After ATP binding, both heads take the ADP.Pi state and diffuse on the actin filament. The two heads alternate strong binding and swing their respective necks. At this time, load is exerted on both heads, resulting in force dependencies on successivity and unidirectionality that decreased two-fold compared to M5SH (Fig. 6D and 7).

The geometry of the acto-myosin interaction is very important for the successive steps taken by myosin-V, as it influences the search-catch behavior made by the myosin-V head. In cells, myosin-V transports cargo toward the pointed end of actin filament, which is the direction of the membrane. Since actin filaments make up a meshwork that regularly overlays filaments on top of one another, myosin-V often switches filaments during its motility. However, such switching is likely inefficient for transport. Assuming the successivity of the myosin-V head depends on the acto-myosin geometry, myosin-V can preferentially select an actin filament without switching. The hand-over-hand mechanism, along with regulating movement, may also regulate the geometry in order to optimize transport.

## Materials and Methods

### Proteins

Actin and myosin rods were obtained and purified from rabbit skeletal muscle [19], [20]. To visualize under an optical fluorescence microscope, actin filaments were labeled with rhodamine-phalloidin (Molecular Probes) and myosin rods with TRITC (Amersham Biosciences, Piscataway, NJ). Recombinant calmodulin from Xenopus oocytes was expressed in Escherichia coli as described [39]. Alpha-actinin was obtained from chicken gizzard and purified [40].

### Recombinant one-headed myosin-V (M5SH) and two-headed myosin-V (M5DH)

Myosin smooth muscle rod having no head (SMrod) and myosin-V subfragment 1 (M5S1) SMrod chimera constructs were produced as previously reported [19], [23]. M5SH heterodimers were made by coinfecting Sf9 cells with viruses expressing M5S1-SMrod fused to His tag, FLAG-SMrod, and calmodulin, respectively, and then purifying both the His and FLAG affinities as previous reported [19]. M5DH was produced by coinfecting Sf9 cells with M5S1 SMrod and calmodulin expressing viruses, respectively, and purified as previously described [19], [23].

### Cofilaments for M5SH and M5DH single molecules

M5SHs were copolymerized into long (6.6 µm on average) filaments with rabbit skeletal muscle myosin rods without heads. The total protein concentration was set to 0.15 µM. The molar ratio of the M5SH to myosin rod in the mixture was adjusted to be 1∶2000 so that only a small number of M5SHs was incorporated into a cofilament [19]. The presence of SMrods in the preparation was not a problem in this assay because M5SH/SMrod cofilaments contained a large excess of skeletal muscle myosin rods. M5DH/SMrod cofilaments were prepared the same way. To visualize cofilaments, a small amount of TRITC-labeled myosin rods were included [20], [23]. The number of the M5SH or M5DH molecules mixed with a cofilament was estimated and discussed in a previous report [19].

### Single-Molecule Mechanical Assay

To attach beads to the two ends of an actin filament, the surface of polystyrene latex beads (0.945 µm in diameter) was coated with α-actinin as previously described [19]. Cofilaments applied to a flow chamber with pedestals on a glass slide surface were adsorbed onto the pedestal surface [19], [20], [23]. The pedestal surface was coated with casein to prevent α-actinin-coated beads from nonspecific binding to the glass surface. An assay buffer (120 mM KCl buffer containing 1–1000 µM ATP, 0.2 mg/ml calmodulin) containing rhodamine phalloidin-labeled actin filaments and α-actinin-coated beads was introduced into the chamber. The actin filament and myosin cofilament were visualized under an epifluorescence microscope. The two ends of an actin filament were attached to optically trapped beads through α-actinin. The suspended actin filament was then brought into contact with a cofilament on the pedestal. Angles between the actin filament and cofilament were chosen to be 65∼80° for M5SH and 45∼60° for M5DH, as these are ideal angles for successive steps [19]. A bright image of a bead, which was captured by optical tweezers and illuminated by a halogen lamp, was projected onto a quadrant photodiode detector. Displacement of the bead was determined with nanometer accuracy [41]. The assay was carried out at 25°C. To reduce photobleaching, an oxygen scavenger system was added to the assay buffer [42]. Position data were obtained at a sampling rate of 24 kHz and filtered and decimated through a 200 Hz Chebyshev filter since the corner frequency of the position measurements was 200 Hz.

## Supporting Information

Figure S1 Single molecular motility assay of GFP (green fluorescent protein) labeled myosin-V. (A) Sequential images of a single myosin-V HMM fused to GFP moving along an actin filament in 1 mM ATP. Green spots are myosin-V HMM; red lines are the actin filament. Scale bar is 500 nm. (B) Histogram of the GFP labeled myosin-V HMM travel distance. (C) Histogram of GFP label myosin-V S-1 (red) travel distance and orthogonal axis (green) along the actin filament. The histograms in B and C were fitted to a single exponential function with travel distances of 250 nm (B, blue line), 98 nm (C, red line) and 58 nm (D, green line), respectively.
(2.66 MB TIF)

Figure S2 A possible working model for M5SH. An explanation is provided in the text. ‘D.Pi’ and ‘D’ indicate ADP.Pi and ADP states, respectively.
(6.06 MB TIF)

Figure S3 A possible working model for M5DH without applying the hand-over-hand mechanism. An explanation of the model is provided in the text. ‘D.Pi’ and ‘D’ indicate ADP.Pi and ADP states, respectively. Red arrows indicate a loaded force; the length of the arrow represents the strength of the load.
(2.37 MB TIF)
