# Supplementary figures and images for: Mechanical Characterization of One-Headed Myosin-V Using Optical Tweezers
Source: PLoS One. 2010 Aug 18;5(8):e12224. doi: 10.1371/journal.pone.0012224 (PMC2923604; doi:10.1371/journal.pone.0012224)

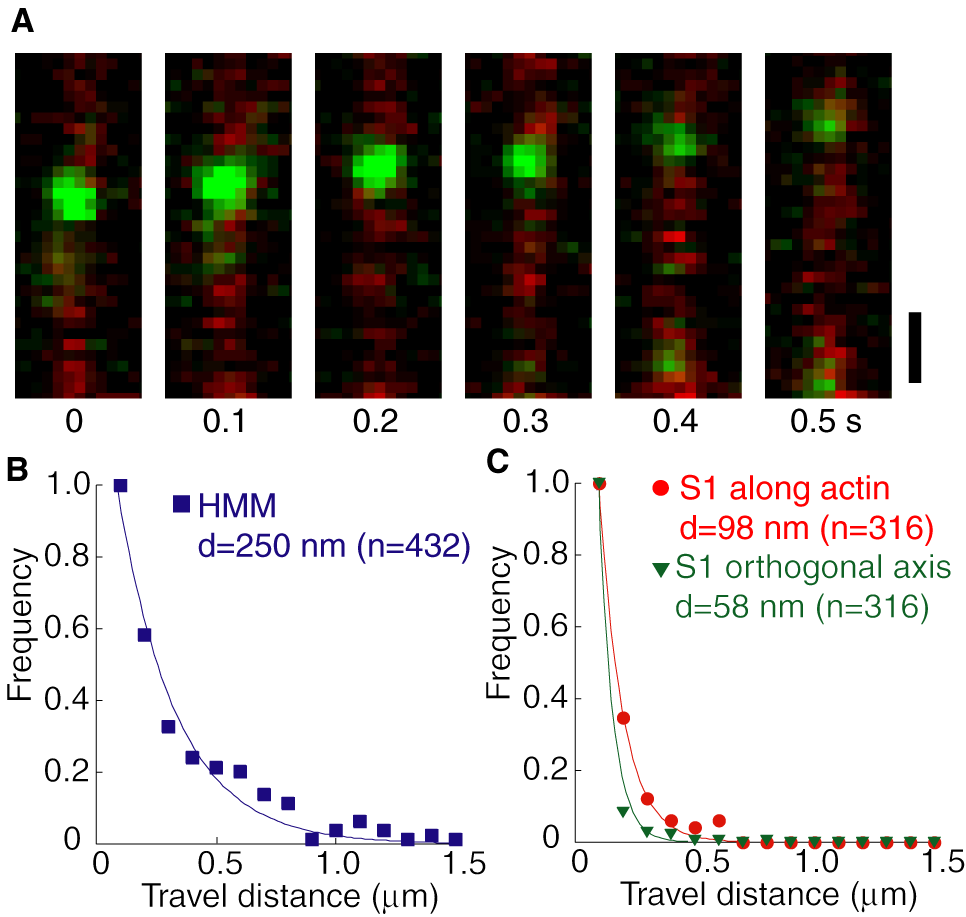

Supplement: Figure S1 — Single molecular motility assay of GFP (green fluorescent protein) labeled myosin-V. (A) Sequential images of a single myosin-V HMM fused to GFP moving along an actin filament in 1 mM ATP. Green spots are myosin-V HMM; red lines are the actin filament. Scale bar is 500 nm. (B) Histogram of the GFP labeled myosin-V HMM travel distance. (C) Histogram of GFP label myosin-V S-1 (red) travel distance and orthogonal axis (green) along the actin filament. The histograms in B and C were fitted to a single exponential function with travel distances of 250 nm (B, blue line), 98 nm (C, red line) and 58 nm (D, green line), respectively. (2.66 MB TIF) [file pone.0012224.s001.tif]

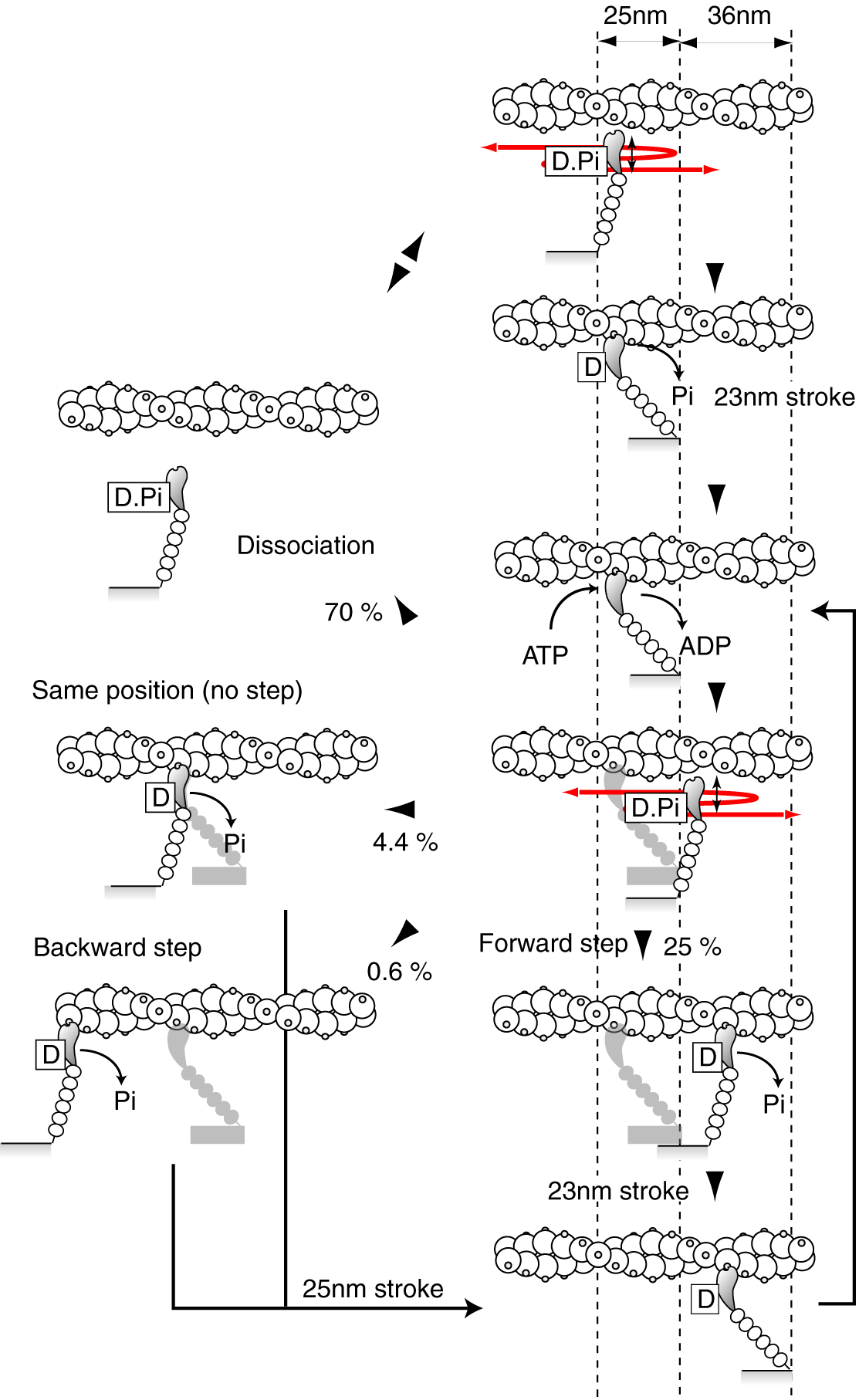

Supplement: Figure S2 — A possible working model for M5SH. An explanation is provided in the text. ‘D.Pi’ and ‘D’ indicate ADP.Pi and ADP states, respectively. (6.06 MB TIF) [file pone.0012224.s002.tif]

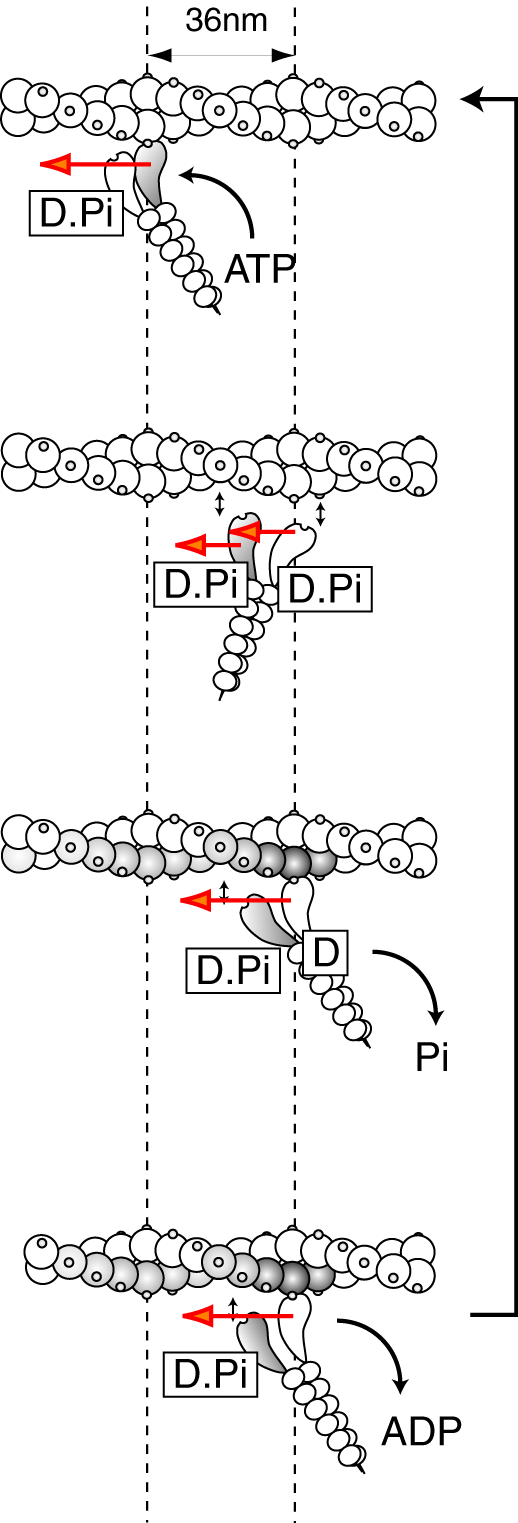

Supplement: Figure S3 — A possible working model for M5DH without applying the hand-over-hand mechanism. An explanation of the model is provided in the text. ‘D.Pi’ and ‘D’ indicate ADP.Pi and ADP states, respectively. Red arrows indicate a loaded force; the length of the arrow represents the strength of the load. (2.37 MB TIF) [file pone.0012224.s003.tif]
